# Examining Change in the Frequency of Adaptive Actions as a Mediator of Treatment Outcomes in Internet-Delivered Therapy for Depression and Anxiety

**DOI:** 10.3390/jcm11206001

**Published:** 2022-10-11

**Authors:** Madelyne A. Bisby, Nickolai Titov, Blake F. Dear, Eyal Karin, Andrew Wilhelms, Marcie Nugent, Heather D. Hadjistavropoulos

**Affiliations:** 1 eCentreClinic, School of Psychological Sciences, Faculty of Medicine, Health, and Human Sciences, Macquarie University, Sydney 2109, Australia; 2 MindSpot Clinic, MQ Health, Macquarie University, Sydney 2109, Australia; 3 Department of Psychology, University of Regina, 3737 Wascana Parkway, Regina, SK S4S 0A2, Canada

**Keywords:** anxiety, depression, iCBT, actions, Internet, treatment, guidance

## Abstract

Adaptive actions, including healthy thinking and meaningful activities, have been associated with emotional wellbeing. The Things You Do Questionnaire—21 item (TYDQ-21) has recently been created to measure the frequency of such actions. A study using the TYDQ-21 found that adaptive actions increased across Internet-delivered therapy for symptoms of depression and anxiety, and higher TYDQ-21 scores were associated with lower psychological distress at post-treatment. The current study examined the relationships between adaptive actions and psychological distress among adults (*n* = 1114) receiving Internet-delivered therapy as part of routine care in Canada, and explored whether adaptive actions mediated reductions in depression and anxiety. As hypothesised, adaptive actions increased alongside reductions in depression and anxiety symptoms from baseline to post-treatment. Treatment effects were consistent when the intervention was provided with regular weekly therapist support or with optional weekly therapist support, and some (but not all) types of adaptive actions had a mediating effect on change in depressive symptoms. The present findings support further work examining adaptive actions as a mechanism of change in psychotherapy, as well as the utility and scalability of Internet-delivered treatments to target and increase adaptive actions with the aim of improving mental health.

## 1. Introduction

An important body of work has previously demonstrated that our thoughts and behaviours, that is, “actions”, can have a significant impact on our psychological health [1,2,3]. More recent work has identified the importance of a broader range of everyday actions such as eating well and sleeping [4,5], as well as those actions which are the target of psychological therapies, like pleasant activities and challenging unhelpful thoughts [6,7,8]. In a recent Danish study, over 3000 respondents reported the daily actions they took to enhance their mental health. A vast range of actions were reported, from which five main themes emerged: positive mindset (e.g., ‘be optimistic’), health behaviours (e.g., ‘exercise daily’), social relationships (e.g., ‘have a social network’), relaxation (e.g., ‘remember to relax’), and use of the brain (e.g., ‘solve tasks’) [9]. Actively doing something to improve mental health was associated with greater self-reported mental wellbeing in the sample. Moreover, approximately half of respondents reported that they had engaged in something ‘to a large extent’ to enhance their mental health within the past two weeks [9]. This finding is consistent with other recent research illustrating a strong association between daily actions and psychological health [10], and highlights the clinical potential of increasing the frequency of such actions to facilitate better psychological health.

The Things You Do Questionnaire—21-item (TYDQ-21) was developed to capture adaptive (i.e., helpful) actions most strongly associated with psychological health [10]. Across two surveys, over 6000 Australian respondents from general community samples reported the frequency with which they engaged in a large range of adaptive actions previously identified in the literature as related to psychological health. Using survey methodology [11], responses were ranked and exploratory factor analysis revealed five dominant factors/categories of adaptive actions: Healthy Thinking, Meaningful Activities, Goals and Plans, Healthy Habits, and Social Connections. The results showed that those who engaged in the identified adaptive actions at least half of the days of the week reported lower symptoms of depression and anxiety, and conversely, increased satisfaction with life. The five domains of actions captured by the TYDQ-21 shared some overlap with the five domains identified by Santini et al. [9], including cognitive actions, health behaviours/routines, and social interactions.

In an extension of this work, a subsequent study examined whether the frequency of these actions changed during Internet-delivered psychological treatment and whether these changes were associated with changes in symptoms [12]. Internet-delivered treatments are increasingly available as a form of mental health care [13], and are as effective as face-to-face treatments for a range of mental health conditions [14,15]. Internet-delivered treatments typically include a series of carefully developed modules alongside therapist support over email, private messaging, or telephone [16]. In that study, 409 individuals reported the frequency of adaptive actions on a fortnightly basis during therapist-guided Internet-delivered cognitive behaviour therapy (ICBT) for depression and anxiety [12]. The five categories of actions first identified by Titov et al. [10] were replicated using confirmatory factor analysis, indicating that the five broad domains of action remained consistent across both community and treatment-seeking samples. The frequency of adaptive actions increased across treatment, and increases in adaptive actions mirrored the decreases in depressive and anxiety symptoms over time. At post-treatment, participants who reported engaging in the actions at least half the days of the week reported significantly lower depressive symptoms, lower anxiety symptoms, and this was the case for all five domains of action [12]. Although the study’s design did not allow causal conclusions, there was an association between increased frequency of adaptive actions and symptom reductions during Internet-delivered psychological treatment for depression and anxiety.

Notably, all participants in that trial received optional therapist guidance—that is, guidance was provided based on participant preference and clinical need. As previous meta-analyses have identified that psychological treatments with ongoing, regular therapist support are more effective than self-guided treatments [17,18], the increase in adaptive actions across treatment may have been, at least for some participants, driven by contact with the therapist, rather than due to the treatment itself. As a result, there are outstanding questions about the role of therapist guidance in the increased frequency of adaptive actions during treatment. Even within the broad construct of ‘therapist guidance’, there are differences in how therapist guidance is provided. Whereas optional guidance involves clinical contact at the behest of the participant and identified clinical need, regular guidance is often provided on a structured, weekly basis and can be initiated by the clinician irrespective of patient preference or clinical need [19,20]. When individuals are randomly allocated to one or the other, participants report equivalent and large reductions in anxiety and depression symptoms at post-treatment and 3-month follow-up [20,21], although treatment completion has been lower in participants receiving optional guidance [20]. In contrast, when individuals receive their preferred guidance option, there are no differences in treatment completion and outcomes [22], highlighting the importance of considering individual preferences when providing therapist guidance. It remains unclear how the frequency of daily actions may change differently based on the type of therapist guidance provided alongside Internet-delivered treatment.

The primary aim of the current study was to extend previous psychometric validations of the TYDQ-21 in Australian samples [10,12] to a Canadian sample of individuals seeking treatment at a digital mental health service. We expected the factor structure previously reported with Australian adults would be replicated in a Canadian sample of adults. We also expected that changes in the frequency of adaptive actions would increase during treatment as symptoms of psychological distress decreased, consistent with past work. We explored whether standard or optional therapist support was associated with different magnitude of changes in psychological symptoms and/or adaptive actions, and replicated previous analyses examining whether participants who reported engaging in adaptive actions at least half the days each week, on average, reported lower depressive and anxiety symptoms.

The secondary aim of the current paper was to examine the contribution of increased frequency of adaptive actions to reductions in psychological symptoms across treatment using mediation. Although not to be taken as conclusive evidence of causality, mediation analyses are an important step towards determining whether a particular variable is a mechanism of treatment change [23,24].

## 2. Materials and Methods

### 2.1. Design

Observational data were collected as part of routine care at the Online Therapy Unit, funded by the Saskatchewan government to provide transdiagnostic ICBT to Saskatchewan residents. The collection of observational data received ethics approval from the University of Regina, and informed consent was obtained from all participants involved in the study.

### 2.2. Recruitment

Participants were recruited between 17 December 2020 and 16 December 2021. Interested persons were directed to the study website (www.onlinetherapyuser.ca, accessed on 1 September 2022) and completed an initial assessment via REDCap. Eligibility was then confirmed during a brief telephone screening interview. Participants were considered eligible if they: (1) were 18 or older; (2) endorsed symptoms of depression and/or anxiety; (3) were Saskatchewan residents and would be in the province for at least 8 weeks; (4) reported access to a computer and the Internet; (5) provided a medical contact for emergency purposes; and (6) had interest in and consented to ICBT. Exclusion criteria included: (1) recent hospitalization, high crisis service involvement, or high risk of suicide; (2) severe alcohol or drug problems; (3) concurrent weekly mental health treatment; (4) seeking help for a different mental health condition; or (5) self-reported medical condition which would interfere with participation. Ineligible applicants were referred to community mental health services in their area.

**Figure 1:**
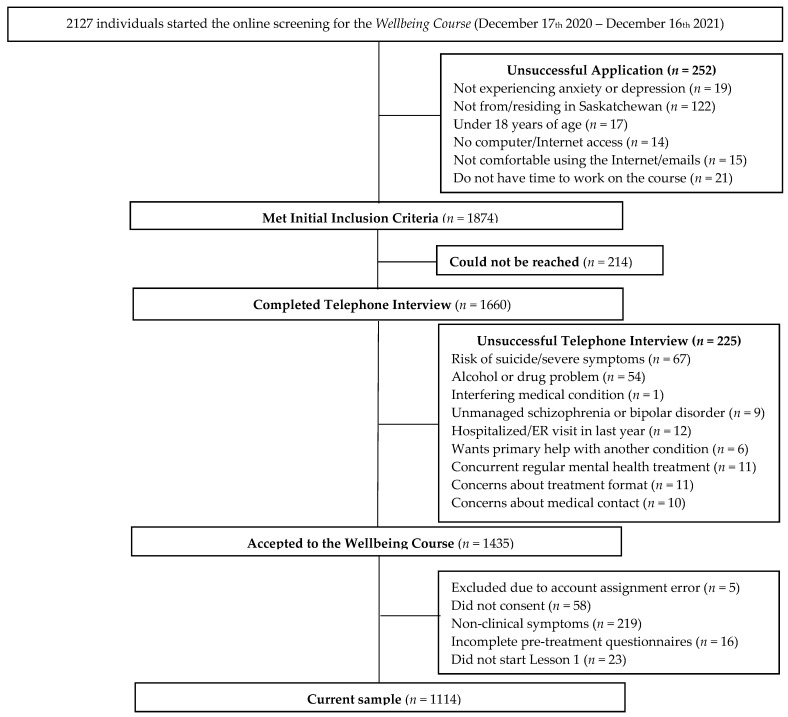
Participant flow chart.

During the recruitment period, 2127 individuals expressed interest and completed an initial assessment, of which 1874 applicants met initial inclusion criteria. The telephone interview was completed by 1660 applicants, and 1435 individuals were accepted into the trial. The current study utilized a subsample of these participants (*n* = 1114) who reported clinically significant symptoms on the depression or anxiety measure (as defined below), completed pre-treatment questionnaires, and began Lesson 1. Participant flow is provided in Figure 1.

### 2.3. Intervention

All participants received access to the Wellbeing Course, a transdiagnostic ICBT course developed by the eCentreClinic at Macquarie University (for further detail, see [25]). This 8-week course consists of 5 core lessons gradually released in a sequential order and cover: (1) psychoeducation; (2) thought challenging; (3) controlled breathing and activity scheduling; (4) graded exposure; and (5) relapse prevention planning and goal setting. An optional booster lesson with a review of content was available one month after post-treatment. Each lesson was accompanied by case stories, additional resources, and a downloadable guide, which includes homework tasks and frequently asked questions. Participants were informed that it would take several hours per week to work on the course (e.g., reading materials and practicing skills). The course was delivered on an individual basis (i.e., not in a group format) and participants progressed through the course at their own pace. In line with previous research, however, participants also received weekly automatic emails to facilitate engagement [26]. These emails encouraged them to work through the course at the following pace: 1 week for Lessons 1 and 3, and 2 weeks for Lessons, 2, 4, and 5. Furthermore, therapist support was only available for 8 weeks, which also encouraged participants to complete the course in a timely manner.

### 2.4. Therapist Guidance

As the current study examined the influence of therapist guidance level on treatment outcome, only those participants with clinical symptoms were included in the current sample to eliminate potential confounds. The level of therapist guidance depended on the participant’s preference, whereby participants chose between ‘optional support’ and ‘standard support’ (see [20] for further details). Each participant was assigned a single therapist for the duration of the course. In the standard support condition, participants were encouraged to contact therapists as often as they liked during the week via email, and therapists replied once per week with the goal to engage participants, reinforce progress, and highlight lesson material where required. In the optional support condition, therapists only contacted participants if they initiated contact. In both groups, therapists contacted participants in the case of symptom deterioration or an increase in risk of suicide/self-harm. Therapists also provided support over the phone where appropriate. All therapists had training in social work or psychology, and were trained and supervised in ICBT.

### 2.5. Measures

#### 2.5.1. Primary Outcome

Things You Do Questionnaire 21-item (TYDQ-21). The 21-item version of the TYDQ-21 described in Study 2 of Titov et al. [10] was used in this study. The list included daily actions associated with psychological wellbeing and consisted of five factors—Healthy Thinking (e.g., treating yourself with respect), Meaningful Activities (e.g., doing something enjoyable), Goals and Plans (e.g., setting achievable goals), Healthy Habits (e.g., regular sleep and wake times), and Social Connections (e.g., spending time with family or friends). A 5-point Likert rating scale asked participants how often they performed actions over the past week using the following scoring system: 0 = “Not at all”; 1 = “One or two days”; 2 = “Half the days”; 3 = “Almost every day”; and 4 = “Every day”. The TYDQ-21 was administered at pre-treatment and post-treatment, and Cronbach’s alpha ranged from 0.91 to 0.94.

#### 2.5.2. Secondary Outcomes

Patient Health Questionnaire 9-item (PHQ-9). The PHQ-9 is a measure of DSM-IV-congruent depressive symptoms over the past two weeks [27,28]. The PHQ-9 has nine items and is scored using a 4-point Likert scale ranging from 0 (“Not at all”) to 3 (“Nearly every day”). A PHQ-9 score of ≥10 was used to identify those with clinical levels of depression [29], although it was recognised that this threshold may over-estimate cases [30]. Cronbach’s alpha in this study ranged from 0.84 to 0.89.

Generalized Anxiety Disorder 7-item (GAD-7). The GAD-7 is a measure of general anxiety symptoms over the past two weeks, designed to detect DSM-IV-congruent generalized anxiety, social anxiety, and panic disorder [31]. The GAD-7 is scored using a 4-point Likert scale ranging from 0 (“Not at all”) to 3 (“Nearly every day”), and a score of ≥10 was used to identify those with clinical levels of anxiety [31,32]. Cronbach’s alpha for this study ranged from 0.87 to 0.91.

### 2.6. Statistical Analysis

Confirmatory factor analyses were conducted in IBM SPSS Amos version 26 and tested the five-factor structure of the TYDQ-21 identified by Titov et al. [10]. Model fit was determined using the comparative fit index (CFI; ≥0.90–0.95), Tucker-Lewis Index (TLI, ≥0.90–0.95), and Root Mean Square Error of Approximation (RMSEA; ≤0.08) [11,33].

All other analyses were conducted using IBM SPSS version 27. The intent-to-treat sample (*n* = 1114) was used for treatment outcome and adherence analyses. Missing data at post-treatment were handled using multiple imputation, accounting for baseline symptom severity and lesson completion [34,35]. Generalized estimating equation (GEE) modelling examined changes over time from pre-treatment to post-treatment [36]. Time and therapist support were entered as predictors. A gamma with log link response scale was used to address skewness within the dependent variables [34], and an unstructured working correlation accounted for different rates of change between participants. Cohen’s *d* effect sizes and 95% confidence intervals were calculated based on estimated marginal means. One-way ANOVAs were used to compare depression and anxiety symptoms between participants based on the frequency of adaptive actions (i.e., above or below half the days in each week, on average, for each domain).

To examine mediation, the PROCESS v4.0 macro was used [37]. Only complete cases were used for mediation analyses (*n* = 751). Supplementary analyses did not find a significant interaction between time, therapist support level, and TYDQ21 scores for change in depression symptoms (*p* = 0.59) or anxiety symptoms (*p* = 0.52). Given the similarities in the two treatment samples, and given the opportunity to increase statistical power, the two therapist support groups were treated as a single group in mediation analyses. Prior to analysis, all variables were log-transformed to ensure that mediation models reflected the percentage change in the dependent variable which was explained by a one-unit percentage increase in the predictor variable (e.g., log-log regression, [38]). Log-transformed pre-treatment scores (PHQ-9 or GAD-7) were entered as the independent variable (X), post-treatment scores (PHQ-9 or GAD-7) as the dependent variable (Y), and post-treatment TYDQ-21 scores as the mediating variable (M). Pre-treatment TYDQ-21 scores were entered as a covariate, consistent with ANCOVA models of mediation [39]. In the case that a mediating effect was detected (i.e., the indirect effect did not include zero), reverse causality models were then used to test the mediating effect. Specifically, reverse causality models tested whether pre-treatment TYDQ-21 scores also mediated change in the dependent variable (as opposed to post-treatment TYDQ-21 scores in the original model). If reverse mediation was found, we concluded that the original mediating effect was not reliable.

## 3. Results

### 3.1. Baseline Demographic and Clinical Characteristics

Participant demographics and symptom severity are presented in Table 1. Participants were mostly female (78.2%), consistent with typical psychotherapy research samples [40,41,42]. The average age of the sample was 37.14 years (range 18–81 years), and this did not differ between guidance conditions (*p* > 0.05). Approximately half were in paid employment (52.1%), and not currently taking medication for their mental health (57.3%), with no differences between guidance conditions (*p*s > 0.05).

In the standard support group, therapists sent an average of 8.35 (SD = 1.61) emails and received an average of 3.01 emails (SD = 2.73) per participant. In the optional support group, therapists sent an average of 4.37 (SD = 2.22) emails and received an average of 2.26 emails (SD = 3.00) per participant.

### 3.2. Confirmatory Factor Analysis

CFAs were used to test the goodness-of-fit of the five-factor model of the TYDQ-21 identified by Titov et al. [10] at pre-treatment and post-treatment. At pre-treatment, the five-factor model showed an acceptable fit to the data: CFI = 0.93, TLI = 0.93, RMSEA = 0.06. Factor loadings ranged from 0.45 to 0.84 (see Supplementary Material, Figure S1). Using complete cases at post-treatment, the five-factor model also showed an appropriate fit: CFI = 0.94, TLI = 0.93, RMSEA = 0.06. Factor loadings at post-treatment ranged from 0.59 to 0.85 (see Supplementary Material, Figure S1).

### 3.3. Treatment Engagement

Participants completed an average of 3.90 (SD = 1.41) lessons in the standard support group and 4.08 (SD = 1.28) lessons in the optional support group, with no significant difference (*p* > 0.05).

### 3.4. Change in Adaptive Actions during Treatment

**Figure 2:**
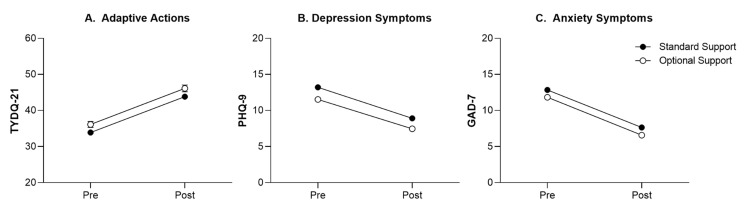
Change in adaptive actions (**A**), depression symptoms (**B**), anxiety symptoms (**C**) from pre-treatment to post-treatment in participants who received standard support and optional support alongside ICBT.

TYDQ-21 Total Score. Overall, participants reported a 29% increase (95% CI 25%, 33%) in the frequency of adaptive actions measured by the TYDQ-21 from pre- to post-treatment (main effect of time, *p* < 0.001; see Table 2 and Figure 2A). Irrespective of timepoint, those participants who received standard support reported engaging in these adaptive actions to a lesser extent than those who received optional support (main effect of guidance, *p* = 0.02; see Table 3), with similar within-group effect sizes (*d*s = 0.65). Consistent with this main effect, there were significant differences in TYDQ-21 scores at pre-treatment (*p* = 0.021) and post-treatment (*p =* 0.04) according to guidance. There was no difference in how TYDQ-21 scores changed from pre- to post-treatment based on guidance condition (time x guidance interaction, *p* > 0.05).

TYDQ-21 Factor Scores. Irrespective of guidance condition, participants reported increases in the frequency of adaptive actions on each of the five factors across treatment (main effect of time, *p*s < 0.001; see Table 2). At the overall level, participants reported increases in Healthy Thinking (*d* = 0.60), Meaningful Activities (*d* = 0.50), and Goals and Plans (*d* = 0.48). Smaller effect sizes were observed for Healthy Habits (*d* = 0.27) and Social Connection (*d* = 0.28). Across timepoints, those participants who received standard support reported lower scores on Healthy Thinking (main effect of guidance, *p* = 0.04), Meaningful Activities (*p* = 0.03), and Goals and Plans (*p* = 0.045), but not Healthy Habits or Social Connections (*p*s > 0.05; see Table 3). No significant time x guidance interactions were observed.

### 3.5. Treatment Outcomes

Depressive Symptoms (PHQ-9). Participants experienced a significant reduction in depressive symptoms from pre- to post-treatment (main effect of time, *p* < 0.001; see Table 3 and Figure 2B). When examined according to guidance condition, depressive symptoms were greater in those who received standard support compared to optional support (main effect of guidance, *p* < 0.001), whereby scores were significantly different between support conditions at pre-treatment (*p* < 0.001) and post-treatment (*p* < 0.001). There was no difference in treatment outcome according to guidance level (time x guidance interaction, *p* > 0.05; see Table 3). Participants in the standard support group reported an average reduction of 33%, while those in the optional support group reported an average reduction of 35%, with similar within-group effect sizes (*d* range 0.77–0.79).

Those participants who reported engaging in adaptive actions over half the days each week, on average, reported lower depressive symptoms. This was the case for the TYDQ-21 total score and each of the five domains (*p*s < 0.001).

Anxiety Symptoms (GAD-7). Participants reported a significant decrease in anxiety symptoms on the GAD-7 from pre- to post-treatment irrespective of guidance condition (main effect of time, *p* < 0.001; see Table 2 and Figure 2C). Those participants who received standard support reported greater anxiety symptoms than those who received optional support (main effect of guidance, *p* < 0.001; see Table 3), such that anxiety symptoms were significantly different at pre-treatment (*p* = 0.003) and post-treatment (*p* = 0.001). There were no differences in change in anxiety symptoms over the course of treatment based on the level of support received (time x guidance interaction, *p* > 0.05). Participants in the standard support group reported an average reduction of 40%, while those in the optional support group reported an average reduction of 44% in anxiety symptoms, with similar within-group effect sizes (*d* range 1.01–1.08).

As was observed for depressive symptoms, those participants who reported engaging in adaptive actions over half the days each week, on average, reported lower anxiety symptoms. This was the case for the TYDQ-21 total score and each of the five domains (*p*s < 0.001).

### 3.6. Mediation of Treatment Outcomes by Adaptive Actions

Change in Depressive Symptoms. As can be seen in Table 4, total scores on the TYDQ-21 did not mediate change in depressive symptoms from pre- to post-treatment. However, three factors of the TYDQ-21 were found to have a small, but significant, mediating effect on change in depressive symptoms: Healthy Thinking (indirect effect = 5%), Meaningful Activities (indirect effect = 4%), and Goals and Plans (indirect effect = 3%).

Change in Anxiety Symptoms. TYDQ-21 scores were not found to have a mediating effect on treatment change in anxiety symptoms when used as a summed total or when examined on a factor level. The mediation models can be seen in Table 4.

## 4. Discussion

The present study sought to further understand the relationship between adaptive actions and symptoms of depression and anxiety during ICBT. The primary aim of the current study was to extend previous psychometric evaluations of the TYDQ-21 to a Canadian sample. The five-factor structure of the measure was replicated in the current study, and the frequency of daily actions increased with Internet-delivered psychological treatment. We compared the change in daily actions across treatment based on whether participants selected to receive standard or optional therapist support. No differences in treatment outcomes were found between guidance conditions, such that both groups demonstrated medium to large reductions (i.e., 33% to 44%) in depressive and anxiety symptoms as well as small to medium increases (i.e., 16% to 34%) in the frequency of adaptive actions. Then, adaptive actions were examined as a mediator of treatment change in depressive and anxiety symptoms—we found that some, but not all, types of daily actions mediated change in depressive symptoms from baseline to post-treatment, but not anxiety symptoms. Taken together, these findings suggest that changes in the frequency of adaptive actions across treatment were related to improvements in psychological distress, and that change in adaptive actions were not related to the amount of therapist support received.

The frequency of adaptive actions captured by the TYDQ-21 increased across treatment, as anticipated. However, a novel finding of the current study was that similar increases in adaptive actions were observed whether participants received regular therapist guidance or only the option of guidance. This was consistent with past work examining the role of therapist guidance in Internet-delivered therapy which report no differences in treatment adherence or outcomes between participants who choose to receive optional or standard guidance [19,21,22]. Notably, over two-thirds (73.7%) of participants chose to receive standard support, and those who selected standard support had more severe depressive and anxiety symptoms and lower TYDQ-21 scores. This finding is similar to previous reports of participants with more severe anxiety symptoms preferring standard support [22], and highlights the importance of considering baseline symptom severity when determining if an individual would benefit from regular, structured therapist guidance. Nevertheless, our findings suggest that therapist guidance may not be essential to support increases in adaptive actions across treatment. However, it should also be noted that all participants in this trial received an interview with a therapist prior to treatment which is likely to increase a participant’s motivation and facilitated engagement and outcomes, and thus this finding may not extend to fully automated ICBT interventions.

The current study also found that participants who reported engaging in adaptive actions most of the time (defined as at least half the days in each week) reported lower depressive and anxiety symptoms. This pattern of findings was consistent for the total TYDQ-21 score as well as each of the five domains of actions, potentially suggesting that all domains of adaptive activity (e.g., healthy thinking, meaningful activities, goals and plans, healthy habits, and social connections) are associated with emotional wellbeing. Unexpectedly, the magnitude of change in adaptive actions was smaller than previously seen in a different study which examined changes in the TYDQ-21 during the same ICBT course. For example, Bisby et al. [12] reported a 45% (95% CI 38, 52) increase in the frequency of actions in the Healthy Thinking domain, whereas the current study reports an overall increase of 33% (95% CI 28, 38) in the same domain, and this pattern is consistent across all five domains of the TYDQ-21. However, the magnitude of improvements in depression and anxiety symptoms are consistent across both the current sample and the clinical trial sample (e.g., 34% [95% CI 30, 38] vs. 37% [95% CI 33, 42] in depression), demonstrating consistency in treatment efficacy. The difference in the change in TYDQ-21 scores may be due to the nature of the participant samples used; whereas Bisby et al. [12] used a sample of participants who received treatment through a specialist research clinic in Australia in 2018-2019, the current study used a sample of participants who received treatment as part of government-funded routine care in Canada in 2020-2021. This comparison illustrates the importance of testing the reliability of results across different settings, countries, and timeframes, but also demonstrates the consistency of the overall patterns (i.e., increased frequency of adaptive actions and reduced psychological distress).

A mediating effect of Healthy Thinking, Meaningful Activities, and Goals and Plans were found on the change in depressive symptoms from pre- to post-treatment. This effect was not observed for anxiety symptoms, suggesting that the adaptive actions captured by the TYDQ-21 domains are more relevant for reductions in depressive, rather than anxiety, symptoms during treatment, at least in this sample. An increase in the frequency of the adaptive actions captured by the Healthy Thinking domain is consistent with the use of key strategies taught in cognitive therapies, such as challenging unhelpful/unrealistic thoughts and treating oneself in a fair and reasonable way [43,44]. Similarly, an increase in the actions captured by the Meaningful Activities domain is consistent with strategies taught in behavioural therapies (e.g., doing something enjoyable), which assert that doing more mastery and pleasure activities will result in improved mood [45,46]. Lastly, an increase in how frequently individuals set goals and plan ahead, as captured by an increased frequency of actions in the Goals and Plans domain, is consistent with a change in perspective from past-focus to future-focus, a process which is thought to be dysregulated in depression [47,48]. Future work may examine the variables which drive increased frequency of different adaptive actions during treatment, and compare the release of relevant course material with increases in different domains of actions (e.g., map the increase in Healthy Thinking scores relative to the release of thought challenging material). It is, however, important to note that the mediating effects were relatively small, and therefore future work is needed to further understand the clinical and practical significance of these findings.

The current finding of a mediating effect of increased adaptive actions on treatment-related change in depressive symptoms is supportive of further experimental investigations into this relationship. Several converging lines of evidence are required to determine if a given variable is a mechanism of treatment change, including mediation, experimental manipulation, a dose–response relationship, and temporality [24,49,50]. Future work may compare participants who are prompted to increase the frequency of such actions on a regular basis to those participants who are not—or, on the contrary, compare participants who are prompted to decrease the frequency of such actions to those who are not. In addition, to determine the temporal relationship in change between daily actions and psychological distress, more regular outcome measurements (e.g., weekly/fortnightly) would be required throughout treatment in future studies.

The findings of the current study should be considered in light of several limitations. First, by virtue of being an observational study from a routine care digital mental health service, no control group was included in the current study. As a result, we could not determine whether the increase in frequency of adaptive actions was treatment-related or would have occurred in the absence of treatment. Future research examining change in adaptive actions over time in a no-treatment control group is warranted. Second, mediation analyses were only conducted on the completer sample due to constraints with data analysis. Considering that 33% of post-treatment data were missing (35% standard, 26% optional), and missingness is more likely in participants who do not complete treatment or report high baseline symptoms [34], it is possible that the results may differ if they had been conducted with fewer missing data. It would also be highly beneficial to measure the TYDQ-21 more often during treatment as well as at follow-up. Third, due to the exploratory nature of the current study, we did not correct for multiple comparisons in our analyses. Lastly, there were baseline differences in anxiety and depression symptoms between participants who received optional versus standard therapist support. This is consistent with previous preference trials using the same design, in which participants who chose optional support reported lower distress than those who chose standard support [22]. It would be worthwhile for future research to compare treatment-related change in adaptive actions in different therapist support conditions when participants have been randomly allocated.

Overall, it is important to note that this study used data collected during 2021, and is likely influenced by the ongoing impacts of the COVID-19 pandemic in Canada at that time. As the current study was conducted within a digital mental health service, data collection was not impeded due to the pandemic. However, the data collected during this period do appear to have been somewhat impacted. Indeed, the within-group changes in depression symptoms were smaller in the current sample than previously observed using the same treatment program [12,41,51]. This may be a result of the ongoing impacts of the pandemic on people’s lives that are less amenable to change via psychological skills, such as reduced employment (note that fewer individuals were in paid employment in the current sample compared to previous samples from the same setting [51]), social isolation, and medical concerns. Therefore, it would be worthwhile for future research to replicate our analyses using participants who are not experiencing the physical, mental, and emotional impacts of a pandemic.

The present findings speak to the potential for adaptive actions as a mechanism and as a target in psychological treatment. Such actions could form the basis of a brief and self-guided treatments focused on promoting the frequency of these actions to reduce psychological distress, and particularly depression. There is substantial public health potential for developing interventions and treatments with simple, practical messages such as increasing how often individuals perform actions during the week—including doing things they find meaningful and fun, and practicing taking perspective. This approach mirrors that which has been taken by governments worldwide to promote physical health, such as eating fruit and vegetables and participating in regular exercise. Future work which develops and rigorously evaluates such treatments within randomised controlled trials offers both theoretical and pragmatic value.

## Figures and Tables

**Table 1:** Baseline demographic and clinical characteristics.

	**Overall**	**Standard Support**	**Optional Support**
	***N* = 1114**	***n* = 821**	***n* = 293**
Age			
Mean (SD)	37.14 (13.17)	36.95 (13.08)	37.63 (13.38)
Gender			
Male	20.3% (226)	19.9% (164)	21.2% (62)
Female	78.2% (871)	78.6% (645)	77.1% (226)
Other	1.5% (17)	1.5% (12)	1.7% (5)
Employment			
Paid employment	52.1% (580)	51.8% (425)	52.9% (155)
Household	16.5% (184)	16.8% (138)	15.7% (46)
Retired	4.0% (45)	3.7% (30)	5.1% (15)
Student	6.6% (74)	6.6% (54)	6.9% (20)
Unemployed	9.2% (102)	9.1% (75)	9.2% (27)
Health concerns	11.6% (129)	12.1% (99)	10.2% (30)
Current medication			
Yes	476 (42.7%)	42.8% (351)	42.7% (125)
No	638 (57.3%)	57.2% (470)	57.3% (168)
Depressive Symptoms			
Minimal (0–4)	6.7% (75)	5.6% (46)	9.9% (29)
Mild (5–9)	21.8% (243)	21.0% (172)	24.2% (71)
Moderate (10–14)	34.6% (386)	32.5% (267)	40.6% (119)
Moderately Severe (15–19)	24.6% (274)	27.2% (223)	17.4% (51)
Severe (20+)	12.2% (136)	13.8% (113)	7.8% (23)
Anxiety Symptoms			
Minimal (0–4)	4.7% (52)	3.8% (31)	7.2% (21)
Mild (5–9)	24.2% (270)	23.8% (195)	25.6% (75)
Moderate (10–14)	32.9% (367)	32.8% (269)	33.4% (98)
Severe (15+)	38.2% (425)	39.7% (326)	33.8% (99)

Note. % (*n*). Chi-square test of significance reported.

**Table 2:** Overall treatment outcomes and estimated marginal means (*n* = 1114).

	Pre	Post	Percentage Change (95% CI)	Within Groups *d* (95% CI)
TYDQ-21 Total Score	34.96 (0.47)	44.95 (0.55)	29 (25, 33)	0.59 (0.50, 0.67)
TYDQ-21 Thoughts Factor	9.68 (0.15)	12.88 (0.17)	33 (28, 38)	0.60 (0.51, 0.68)
TYDQ-21 Activity Factor	6.39 (0.11)	8.39 (0.13)	31 (26, 37)	0.50 (0.41, 0.58)
TYDQ-21 Goals Factor	7.78 (0.15)	10.29 (0.16)	32 (27, 38)	0.48 (0.40, 0.57)
TYDQ-21 Habits Factor	5.16 (0.10)	6.05 (0.10)	17 (12, 23)	0.27 (0.18, 0.35)
TYDQ-21 Social Factor	5.95 (0.11)	6.97 (0.11)	17 (12, 22)	0.28 (0.19, 0.36)
PHQ-9	12.34 (0.19)	8.15 (0.19)	34 (30, 38)	0.66 (0.58, 0.75)
GAD-7	12.32 (0.17)	7.09 (0.17)	42 (39, 46)	0.92 (0.83, 1.01)

Note. Mean and standard error reported. Between-groups effect size at post-treatment reported. CI = Confidence Interval.

**Table 3:** Treatment outcomes and estimated marginal means according to guidance condition (*n* = 1114).

	Standard Support				Optional Support				
	Pre	Post	Percentage Change (95% CI)	Within Groups *d* (95% CI)	Pre	Post	Percentage Change (95% CI)	Within Groups *d* (95% CI)	Between Groups *d* (95% CI)
TYDQ-21 Total	33.87 (0.47)	43.83 (0.59)	29 (25, 34)	0.65 (0.55, 0.75)	36.10 (0.84)	46.10 (0.95)	28 (21, 35)	0.65 (0.48, 0.82)	0.14 (0.00, 0.27)
TYDQ-21 Thoughts	9.39 (0.15)	12.60 (0.19)	34 (29, 39)	0.65 (0.55, 0.75)	9.97 (0.26)	13.16 (0.28)	32 (24, 40)	0.69 (0.52, 0.86)	0.11 (−0.03, 0.24)
TYDQ-21 Activity	6.17 (0.11)	8.15 (0.13)	32 (27, 38)	0.57 (0.47, 0.67)	6.63 (0.11)	8.63 (0.22)	30 (23, 37)	0.67 (0.50, 0.84)	0.13 (−0.01, 0.26)
TYDQ-21 Goals	7.56 (0.15)	9.95 (0.18)	32 (26, 38)	0.50 (0.40, 0.60)	8.01 (0.26)	10.65 (0.29)	33 (23, 43)	0.56 (0.39, 0.72)	0.14 (0.00, 0.27)
TYDQ-21 Habits	4.98 (0.10)	5.93 (0.11)	19 (13, 25)	0.32 (0.22, 0.41)	5.34 (0.17)	6.18 (0.17)	16 (7, 25)	0.29 (0.13, 0.45)	0.08 (−0.05, 0.21)
TYDQ-21 Social	5.77 (0.11)	6.79 (0.11)	18 (12, 23)	0.32 (0.23, 0.42)	6.14 (0.19)	7.16 (0.19)	17 (8, 25)	0.31 (0.15, 0.48)	0.12 (−0.02, 0.25)
PHQ-9	13.21 (0.19)	8.90 (0.20)	33 (29, 37)	0.77 (0.67, 0.87)	11.52 (0.30)	7.46 (0.30)	35 (28, 42)	0.79 (0.62, 0.96)	0.26 (0.12, 0.39)
GAD-7	12.84 (0.17)	7.64 (0.19)	40 (37, 44)	1.01 (0.90, 1.11)	11.82 (0.29)	6.57 (0.28)	44 (38, 51)	1.08 (0.90, 1.25)	0.20 (0.07, 0.34)

Note. Mean and standard error reported. Between-groups effect size at post-treatment reported. CI = Confidence Interval.

**Table 4:** Mediation of change in symptoms from pre-treatment (X) to post-treatment (Y) using TYDQ-21 post-treatment scores (M; *n* = 751).

Mediator (M)	Path a (X→M)	Path b (M→Y)	Path c (Total Effect X→Y)	Path c’ (Direct Effect X→Y)	Indirect Effect	Reverse Indirect Effect	Conclusion
PHQ-9 (Depressive Symptoms)							
TYDQ-21 Total	−2 ***	−80 ***	76 ***	74 ***	2 (−3, 6)	--	NS mediation
TYDQ-21 Thoughts	−7 *	−72 ***	78 ***	73 ***	5 (0, 9)	3 (0, 5)	Mediation
TYDQ-21 Activity	−8 *	−57 ***	80 ***	76 ***	4 (1, 8)	0 (−3, 3)	Mediation
TYDQ-21 Goals	−7 *	−49 ***	78 ***	75 ***	3 (0, 6)	2 (0, 5)	Mediation
TYDQ-21 Routine	−4	−51 ***	78 ***	76 ***	2 (−1, 5)	--	NS mediation
TYDQ-21 Social	−4	−42 ***	78 ***	76 ***	2 (0, 4)	--	NS mediation
GAD-7 (Anxiety Symptoms)							
TYDQ-21 Total	0	−87 ***	60 ***	59 ***	0 (−6, 5)	--	NS mediation
TYDQ-21 Thoughts	−4	−89 ***	57 ***	53 ***	4 (−3, 9)	--	NS mediation
TYDQ-21 Activity	−1	−62 ***	62 ***	62 ***	0 (−5, 6)	--	NS mediation
TYDQ-21 Goals	−1	−56 ***	62 ***	61 ***	0 (−5, 6)	--	NS mediation
TYDQ-21 Routine	0	−52 ***	62 ***	63 ***	0 (−5, 4)	--	NS mediation
TYDQ-21 Social	0	−37 ***	62 ***	62 ***	0 (−2, 3)	--	NS mediation

* *p* < 0.01, *** *p* < 0.001. NS = non-significant. Note. All mediation models included pre-treatment scores as a covariate. All paths report the percentage change in the dependent variable caused by a one-unit percentage change in the independent variable.

## Data Availability

Not applicable.

## Supplementary Materials

The following are available online at https://www.mdpi.com/article/10.3390/jcm11206001/s1, Figure S1. Confirmatory factor loadings at assessment (left) and post-treatment (right).
