# Supplementary figures and images for: Examining Change in the Frequency of Adaptive Actions as a Mediator of Treatment Outcomes in Internet-Delivered Therapy for Depression and Anxiety
Source: J Clin Med. 2022 Oct 11;11(20):6001. doi: 10.3390/jcm11206001 (PMC9605214; doi:10.3390/jcm11206001)

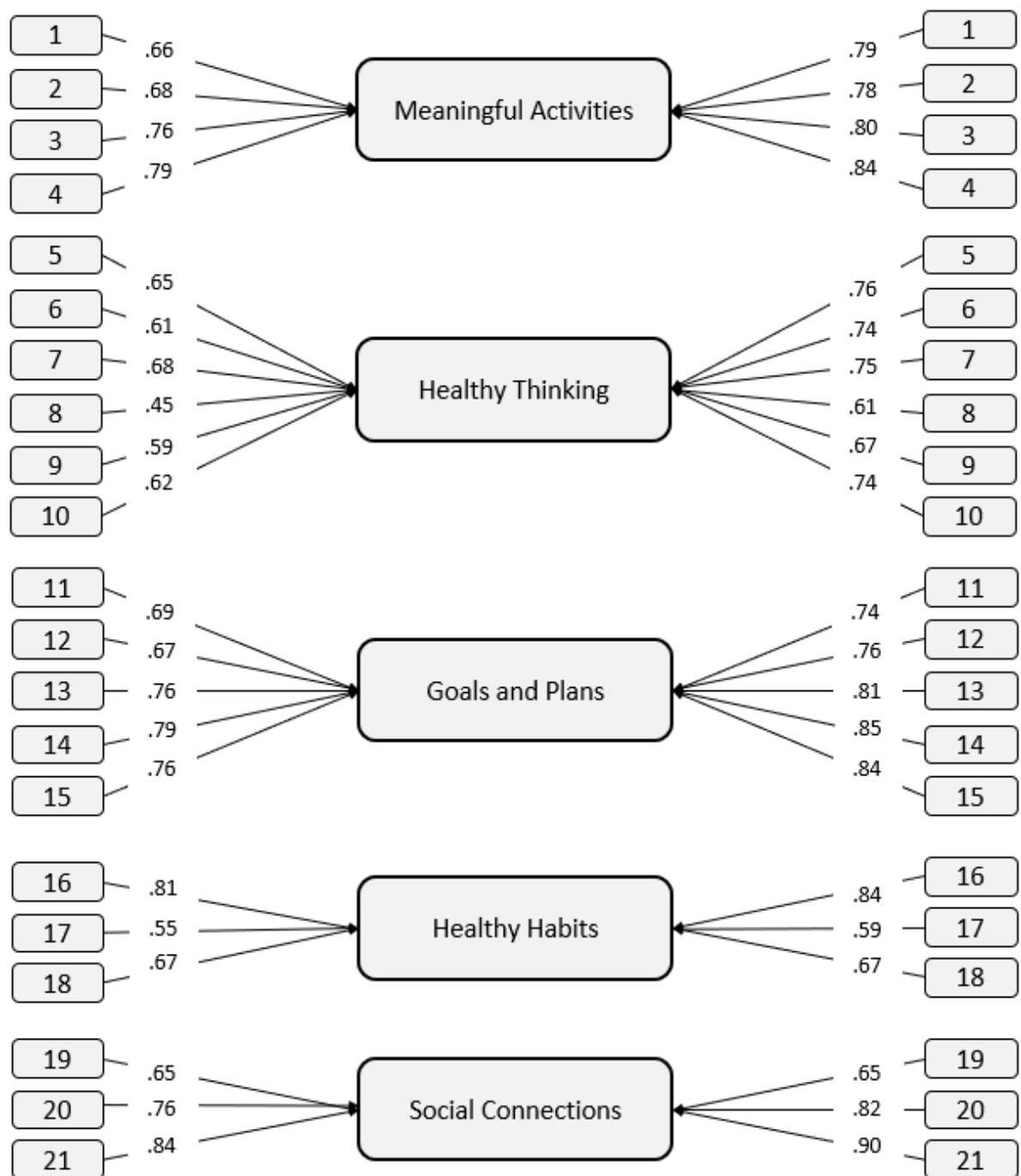

**Figure S1.** Confirmatory factor loadings at assessment (left) and post-treatment (right).

Supplement: Supplementary file 1 [file jcm-11-06001-s001.zip › jcm-1930560-supplementary.pdf]
